# Antecedents and Consequences of Child Externalizing Problems: Differences in Dynamic Parent–Child Processes

**DOI:** 10.1007/s10802-023-01045-0

**Published:** 2023-03-14

**Authors:** Jennifer A. Somers, Kelsey Stiles, Gabrielle A. MacNaughton, Sara J. Schiff, Yixuan Shen, Steve S. Lee

**Affiliations:** Department of Psychology, University of California, Los Angeles, USA

**Keywords:** Parent–child dynamics, Externalizing problems, Dynamic structural equation model

## Abstract

Given that noncompliance is the most common externalizing problem during middle childhood and reliably predicts significant conduct problems, innovations in elucidating its etiology are sorely needed. Evaluation of in-the-moment antecedents and consequences of child noncompliance improves traction on this goal, given that multiple theories contend that child noncompliance and parent behavior mutually influence each other through negative reciprocation as well as contingent praise processes. Among a sample of 140 families (child age: 6–10 years; 32.1% female), the present study capitalized on intensive repeated measures of observed child noncompliance and parent negative talk and praise objectively coded during three unique tasks. We employed dynamic structural equation modeling to evaluate within-dyad parent–child behavioral dynamics and between-dyad differences therein. Results provided mixed support for hypotheses and suggested that antecedents and consequences of child noncompliance differed according to task demands and child ADHD symptoms. Contrary to models of coercive cycles, during child-led play, parent negative talk was more likely following prior child noncompliance, but child noncompliance was less likely following prior parent negative talk. As expected, during parent-led play, parent praise was less likely following prior child noncompliance, which was also less likely following prior parent praise. Relative to youth with fewer symptoms, for children with elevated ADHD symptoms, during a challenging clean-up task, child noncompliance was less stable and less contingent on prior parent negative talk. Results are discussed in terms of their implications of real-time parent–child interactions for typical and atypical development of externalizing problems.

**Supplementary Information:**

The online version contains supplementary material available at 10.1007/s10802-023-01045-0.

Children’s noncompliance with parental requests represents the most common externalizing problem for which parents seek child mental health services (Kalb & Loeber, 2003; Owen et al., 2012). Temporally, parents’ *immediate* response to their child’s noncompliance, including escalating negativity or withdrawal of praise, may reinforce or momentarily resolve behavior problems, thus implicating unique *within-family* processes in the development of child externalizing disorders, including attention-deficit/hyperactivity disorder (ADHD) and disruptive behavior disorders (Granic & Patterson, 2006). To advance etiological theories of youth externalizing psychopathology, elucidating the temporal course of youth noncompliance during typical parent–child interactions is essential. Capitalizing on intensive repeated measures of observed child noncompliance and parent behavior across diverse task demands, the present study employed dynamic methods to rigorously characterize *within-dyad* parent–child behavioral dynamics, including the extent to which children’s noncompliance is both an antecedent of and response to changes in their own parent’s behavior.

Transactional models of development and dynamic systems theories underscore that parent–child interaction *processes* shape developmental trajectories of youth externalizing disorders (Granic & Patterson, 2006). Applied to coercion theory (Patterson, 2002), a dynamic systems lens underscores that moment-to-moment reciprocation and escalation of aversive behaviors between parent and child eventually culminate in parent capitulation to child noncompliance (Granic & Patterson, 2006). Over time, parent capitulation negatively reinforces child noncompliance, which may entrench a stable pattern of externalizing behavior problems (Granic & Patterson, 2006; Lunkenheimer et al., 2016; Patterson, 2002). Although coercive processes and other negative parent–child interaction factors (e.g., inconsistent discipline) are featured more centrally in etiological models of externalizing disorders, positive parenting practices also uniquely predict youth externalizing problems (McFayden-Ketchum et al., 1996). For example, according to theories of emotion socialization, when parents contingently respond to children with warmth and support, children learn to correctly anticipate appropriate affective responses and effectively self-regulate, which may promote their persistence with undesirable tasks and resolve conflict, eliciting more positive parent behavior (Morris et al., 2007). Conversely, children’s negativity may be less likely to elicit supportive parenting, and parents’ positive affect or supportiveness may fail to dampen their children’s negativity when children experience inconsistent caregiving (Granic & Lougheed, 2016; Lougheed et al., 2015).

## Within-Family Processes and Child Noncompliance

Although theories of externalizing development have increasingly emphasized the role of reciprocal, within-dyad parent–child dynamics (e.g., Granic & Patterson, 2006), there is limited empirical evidence on unique within-family processes that govern how child noncompliance unfolds during real-time interactions. Studies of parent–child interactions among youth at risk for externalizing problems typically employ observational methods that rate global parent–child characteristics (e.g., rates of child noncompliance or parent negativity across entire tasks; Li & Lee, 2012; Tung et al., 2015). However, global coding precludes strong tests of *within-dyad* behavioral contingencies occurring *during* parent–child interactions that influence children’s externalizing problems (e.g., noncompliance) *in the moment*. To advance research on parent–child behavioral *processes* implicated in child externalizing problems, elucidation of *moment-to-moment, within-dyad processes during salient tasks* must be prioritized.

Newer dynamic systems approaches have generated empirical evidence that temporally-contingent changes in parent behavior in response to their child’s negativity/noncompliance are implicated in risky trajectories toward externalizing disorders (Granic & Lougheed, 2016; Granic & Patterson, 2006; Lougheed et al., 2015). Compared to typical parents, mothers who endorsed more hostility and elevated child externalizing problems were 35% more likely to change their behavior in response to their preschooler’s off-task behavior during a challenging puzzle task (Lunkenheimer et al., 2016). Parents who reported lower self-regulation were more likely to transition into negative parenting (i.e., negative directives or disengagement) specifically in response to toddler noncompliance during clean-up (Geeraerts et al., 2021). Dynamic models were also applied to positive parent–child processes that may promote child compliance, even during challenging tasks, although empirical evidence is mixed. Stronger contingencies between maternal autonomy support (e.g., guiding a child through tasks, proactively structuring engagement) and child compliance/persistence were related to better child behavioral regulation and fewer child behavior problems (Lunkenheimer et al., 2013, 2017). However, other studies have not observed contingencies between maternal autonomy support and preschool child compliance in negative and neutral contexts (Lobo & Lunkenheimer et al., 2020).

The extant literature faces methodological limitations leaving unanswered questions about for whom and in what contexts reciprocal, dyadic processes governing changes in child noncompliance unfold. The use of methods that preclude the identification of specific within-dyad *antecedents and consequences* of *changes in child externalizing problems (e.g., noncompliance)* limits our understanding of how child noncompliance is organized within the dyad. Despite the assertion that parent–child dynamics are “a function of reciprocal causality unfolding in real time” (Granic & Patterson, 2006, p. 106), most dynamic systems studies focus on modeling survival processes (e.g., time to event; e.g., Granic & Lougheed, 2016; Lougheed et al., 2015), in contrast to *bidirectional* relations between dynamic fluctuations in parent behavior and child noncompliance that unfold over the course of an interaction. Models that account for multiple parent behaviors (e.g., negativity or praise) are also needed to comprehensively elucidate dynamic processes involving momentary child externalizing behavior. Task demands must too be explicitly considered given the effects of dyadic affective contingencies may vary according to demands (Lobo & Lunkenheimer, 2020). Further, evaluations of parent–child interactions often aggregate across interactions; failure to disentangle between-dyad differences in interaction quality from within-dyad variability across tasks may bias results (Roesch et al., 2010). Last, previous dynamic systems work on externalizing behavior focuses almost exclusively on preschoolers; however, parent–child relationships in middle childhood predict child externalizing problems (Pinquart, 2017), which increase prior to adolescence (Loeber & Burke, 2011), making middle childhood a critical period to evaluate parent–child dynamic processes before they are consolidated and catalyze externalizing disorders.

## Current Study

The current study sought to address key knowledge gaps of within-dyad processes governing children’s externalizing behaviors as they unfold during naturalistic parent–child interactions. We evaluated within-dyad, moment-to-moment fluctuations in observed child noncompliance and parent negativity and praise, derived from contiguous 10-s intervals from three discrete, salient, and ecologically valid tasks, among school-aged children with and without ADHD. Using a novel methodological approach, dynamic structural equation modeling (DSEM; Asparouhov et al., 2018), we rigorously examined bidirectional within-dyad, moment-to-moment *augmentation or blunting* of one dyad member’s behavior by their partner’s prior behavior, while simultaneously accounting for the *frequency* and *carryover* (or stability) in an individual’s behavior. Consistent with dynamic systems theory and prior evidence of carryover in child noncompliance (Williams & Forehand, 1984), we hypothesized that, on average, (1a) there would be positive carryover of child noncompliance from one moment to the next (10 s later). Drawing on prior theoretical and empirical work on coercion (Granic & Patterson, 2006; Patterson, 2002) and positive parenting (e.g., Lunkenheimer et al., 2017; Owen et al., 2012), we expected that, on average, there would be within-dyad cross-lagged processes, such that (1b) there would be amplifying effects between parent negative talk and child noncompliance, and (1c) there would be dampening effects between parent praise and child noncompliance from one moment to the next, even after adjusting for within-person frequency and carryover. Although child noncompliance *influences* and is *influenced by* parent behavior (e.g., negativity or praise), the magnitude of these influences may not be equivalent. Disambiguating the *lead-lag structure* of bidirectional cross-lagged processes allows us to separately identify the *antecedents and consequences* of dyadic processes involving child noncompliance. We evaluated these hypotheses separately for each task, as parent socialization goals and the effects of socialization behaviors vary based on situational demands. For example, parents may seek to encourage appropriate regulatory responses in emotionally- and cognitively-demanding situations (e.g., shifting from playing with toys to cleaning up) while scaffolding problem-solving approaches to unexpected challenges in other situations (e.g., repairing a toy that breaks during play; Eisenberg et al., 1998). Given the lack of extant research in this area, we did not have a priori hypotheses about context-specific processes.

Second, because within-family processes are concurrently and prospectively associated with youth externalizing disorders (Lunkenheimer et al., 2015, 2017), we tested between-dyad differences in these within-dyad processes among a sample of children with and without ADHD symptoms. Consistent with the vast literature demonstrating that the traits underlying major child psychiatric disorders are continuous in nature rather than qualitatively distinct shifts from typical functioning (Beauchaine et al., 2018), and specific evidence that ADHD symptoms are associated with psychopathology symptoms across the general population and among individuals both with and without a diagnosis of ADHD (Orm et al., 2022), we adopted a dimensional approach to evaluating between-dyad differences in within-dyad antecedents and consequences of child noncompliance. In addition to the elevated risk for noncompliance among children with elevated disruptive behavior problems and ADHD symptoms (Kalb & Loeber, 2003), we evaluated whether (2) within-child carryover in noncompliance and within-dyad cross-lagged processes involving child noncompliance differed according to child externalizing behavior problems or ADHD symptoms.

## Methods

### Participants

The sample consisted of 140 six- to ten-year-old children (M_age_ = 7.9 years; SD _age_ = 1.1 years) and caregivers participating in a laboratory visit as part of a larger study on youth with and without ADHD and for whom microcoded parent–child interaction data were available. As 99% of caregivers were the child’s parents, we hereafter refer to caregivers as parents; 85.7% of parents identified as female. The sample was racially and ethnically diverse: 54% of children were White, 27.3% were multiracial, 8.6% were Black, 7.2% were Hispanic, and 1.4% were Asian. Sample characteristics are presented in Table 1.

**Table 1:** Sample Characteristics

	ADHD (N = 65)	Non-ADHD (N = 75)
Child Age—M*(SD)*	7.8 *(1.1)*	8.0 *(1.2)*
Child Sex – % Male	69.2%	66.7%
Child Race-Ethnicity – % White	46.9%	60.0%
Child Lives With Siblings %	33.1%	50.9%
Child ADHD symptoms – M(*SD*)	9.1 *(3.1)*	3.5 *(3.1)*
Child disruptive behavior problems – M(*SD*)	14.7 *(9.3)*	6.0 *(6.0)t*
Parent Study Participant – % Mother	86.2%	85.3%
Parent Age*—*M*(SD)*	40.7 *(7.9)*	42.6 *(5.2)*
Parent Race-Ethnicity – % White	73.9%	62.1%
Household Income – % above $60,000	72.6%	70.7%

### Procedures

Families were recruited from mental health centers, pediatric offices, and through flyers posted in local elementary schools and public locations. Inclusion criteria included English fluency, residing with at least one biological parent at least half of the time, and full-time school enrollment. Exclusionary criteria were an IQ below 70 or a neurological, pervasive developmental, or seizure disorder. Study eligibility was based on a telephone screening with the caregiver. Eligible families were invited for a laboratory-based assessment and were mailed rating scales to complete. To capture the full range of functioning in the sample, including differences secondary to the child’s medication status, parents were asked to report on their child’s unmedicated behavior, if possible (e.g., a child who takes stimulant medication on weekdays but not on weekends). Parents were also asked to have their child abstain from medication on the day of the assessment; however, this was not an exclusion criterion if suspending medication was otherwise undesirable or unsafe for the child. Approximately 85% of children were assessed in the lab on days when they had not taken any medication. After obtaining parental consent and child assent, parents and children participated in all activities.

The lab visit lasted approximately four hours and included structured parent–child interaction tasks (Eyberg et al., 2005) that previously demonstrated predictive validity and sensitivity to interventions (Thomas & Zimmer-Gembeck, 2007). Other tasks included neuropsychological and computerized assessments plus parent- and self-reports on children’s symptoms. Multiple breaks were offered to support children’s task engagement. Families were instructed to engage in three tasks in a fixed order: a) a 10-min child-led play, b) a 10-min parent-led play, and c) a 5-min parent-led clean-up time. Families received $50 compensation. The University of California, Los Angeles IRB approved all study procedures prior to study participation.

### Measures

#### Micro-Coded Behavior During Parent–Child Interactions

All parent–child interaction tasks were digitally recorded and coded using the Dyadic Parent Child Interaction Coding System (DPICS; Eyberg et al., 2005), which previously demonstrated moderate to high interrater and test–retest reliability (Chronis-Tuscano et al., 2008). Parent and child behaviors were coded in 10-s intervals, yielding 60 epochs during each 10-min play episode and 30 epochs during the 5-min clean-up. Research assistants completed intensive training on the DPICS until at least 70% agreement with training videos was attained. Weekly coding meetings prevented rater drift and resolved disagreements. To estimate reliability, 20% of the videos were randomly selected and coded by two independent coders. In this study, the intraclass correlations (ICC) for composite categories indicated good reliability (ICC negativity = 0.75; ICC praise = 0.88; ICC noncompliance = 0.78). Because nearly all (> 97%) parent and child variables in each interval were either 0 or 1 (i.e., multiple instances of behavior rarely occurred in a single 10-s interval), variables were dichotomized.

##### Child Noncompliance

Child noncompliance was coded when a child failed to comply with a parental command or when a child failed to respond to a parental request for information (i.e., 1 = noncompliance, 0 = compliance). If the parent did not give a command or asked a question that required a response during that interval, the child’s behavior was coded as missing.

##### Parent Behavior

Parent negative talk was coded when a parent made hostile or critical comments directed toward their child (e.g., “You’re doing that wrong”), negative commands (e.g., “Stop doing that!”), or sarcastic and condescending remarks (e.g., “You think you’re so clever, don’t you?”). Parent praise was coded when they positively appraised their child’s behavior, attribute, or a product created by the child (e.g., “You’re a good builder”).

#### Parent-Reported Child Externalizing Behavior Problems

Parents rated child behavior problems using the 113 item Child Behavior Checklist (CBCL) rating scale (Achenbach & Rescorla, 2001). Items were rated on a 3-point scale (0 = *Not True*, 1 = *Somewhat or Sometimes True*, 2 = *Very True or Often True*) based on the last six months. The validity and reliability of the syndrome and *DSM*-oriented scales were well-established (Achenbach & Rescorla, 2001; Achenbach et al., 2003). As suggested by the scale developer to maximize variance in key variables (Achenbach & Rescorla, 2001), all analyses employed total CBCL subscale scores. Child disruptive behavior problems were estimated from the 35-item CBCL broadband externalizing problems subscale, which included aggressive and rule-breaking behaviors (α = 0.92). Child ADHD symptoms were estimated from the 7-item *DSM*-oriented Attention Deficit Hyperactivity Problems (ADHD) clinical scale, which consists of the seven items most consistent with DSM inattention and hyperactivity-impulsivity (α = 0.88).

## Data Analysis Plan

Three sets of dynamic structural equation models (DSEM; Asparouhov et al., 2018) evaluated within-dyad processes that were allowed to vary between dyads, during child-led play, parent-led play, and clean-up tasks. Primary analyses were conducted using M*plus* (M*plus* v.8.4; Muthén & Muthén; 1998–2017), which uses Bayesian Markov chain Monte Carlo (MCMC) with a Gibbs sampler. We used two unthinned chains, each running for a maximum of 100,000 iterations, to ensure the estimation was stable. We allowed the algorithm to terminate prematurely if the potential scale reduction factor dropped below 1.05 (Gelman & Rubin, 1992). We used the default diffuse prior distributions in M*plus*, which was reasonable given the sample size. Posterior distributions were summarized with the median. In Bayesian analyses, missing data are treated as unknown parameters, which implies that missing data are sampled from their conditional posterior, and MCMC estimation yields consistent estimates when missing data are missing at random (Hamaker et al., 2018).

Binary variables are accommodated in DSEM through the probit link function (Asparouhov & Muthén, 2019). Lagged variables of child noncompliance and parent negative talk and praise were created in M*plus*. The continuous processes underlying the lagged (lag-1) binary predictors were latent centered to yield pure within effects (Hamaker & Grasman, 2015). As a result, the intercept is an unconditional probability that refers to when a person is at their typical, trait-like value for the underlying process of the predictor at time *t*-1 (hereafter referred to as the behavior, or specifically, noncompliance, negative talk, or praise).

At the within-dyad level, the behavior is mean-reverting, such that at any moment, a person may exhibit a state-like fluctuation that is either higher or lower than their trait-level of the behavior. State-like fluctuations in child and parent behavior were predicted by fluctuations in their own prior behavior and each other’s prior behavior during the immediately preceding 10-s epoch. That is, all autoregressive and cross-lagged paths in the within-level model were estimated. Probit models relate the predictors to the outcome through the standard normal cumulative distribution function; thus, regression coefficients correspond to changes in the *Z*-scores associated with the predicted probability.

Random effects were placed on intercepts and slopes of children’s and parents’ behavior at the within-level, which allowed these effects to differ at the between-level. To evaluate Aim 2, models included between-level predictors (grand mean centered child ADHD symptoms or disruptive behavior problems) of the within-level intercepts and autoregressive effects (i.e., carryover effects) and the cross-lagged effects between parents’ and their child’s behavior. No between-level predictors of cross-lagged effects between parents’ behavior were specified. Unstandardized estimates are presented for all models. Similar to a frequentist framework, effects were considered non-null if the 95% credible intervals (CIs) excluded zero. Between-level child predictor effects on within-level relations were probed at the mean and ± 1 SD of the mean on the predictor using a multilevel moderation web utility (Preacher et al., 2006).

## Results

### Preliminary Analyses

We observed one outlier (> 3 SD from the mean) on child disruptive behavior problems. Because results did not change with or without its inclusion, the outlier was not excluded from the dataset, and results are based on all available data. Skewness and kurtosis of all continuous variables (child disruptive behavior problems, child ADHD symptoms; parent and child age) met assumptions of normality (Brown, 2006).

Table 2 presents the overall prevalence of observed child noncompliance, parent negative talk, and parent praise, per task, and results of within-dyad tetrachoric correlations. Given the null correlations in the underlying processes giving rise to these behaviors, as well the large sample size requirements for reliably estimating random effects covariances (Schultzberg & Muthén, 2018), covariances between random effects were not included in primary analyses.

**Table 2:** Overall prevalence of within-dyad behavior and average within-dyad tetrachoric correlations, per task

	Prevalence (%)	1	2
Child-led play			
1. Child Noncompliance	12.14	–-	
2. Parent Negative Talk	3.48	-0.05	–-
3. Parent Praise	4.85	0.01	-0.01
Parent-led play			
1. Child Noncompliance	13.37	–-	
2. Parent Negative Talk	7.52	0.03	–-
3. Parent Praise	6.46	0.01	-0.00
Clean-up			
1. Child Noncompliance	14.89	–-	
2. Parent Negative Talk	8.34	0.03	–-
3. Parent Praise	6.21	0.09	0.00

Results of the tetrachoric correlations are summarized with the posterior distribution medians. If the 95% credible interval contained 0, the effect is considered null (i.e., non-significant); null effects are shown in plain text

Based on bivariate Pearson correlations, independent samples *t*-tests, and one-way ANOVAs, children’s disruptive problems and ADHD symptoms were unrelated to the potential covariates: whether the child lived with siblings; parent and child sex, age, and race-ethnicity; and parent-reported family income (all *p*’s > 0.05). Because they were not related to values of or missingness on primary study variables, these potential between-level covariates were not included in primary analyses.

Children’s disruptive problems and ADHD symptoms were correlated, *r*(137) = 0.718, *p* < 0.001. Child ADHD symptoms and disruptive behavior problems were evaluated in separate models due to multicollinearity concerns.

### Primary Analyses

We first estimated the average within-dyad processes, in each task, with three DSEMs. These models allowed within-dyad dynamics to differ across families but did not include between-dyad predictors. Model-derived estimates of within-dyad intercepts and regression path intercepts, for each task, are shown in Table 3. Supplementary Table 1 presents estimated within-dyad probabilities of child and parent behavior, per task.

**Table 3:** Within-Level Base Model

Within-level intercepts and regression paths							
		Child-led Play		Parent-led Play		Clean-Up	
Effect		Posterior Median	95% Credible Interval	Posterior Median	95% Credible Interval	Posterior Median	95% Credible Interval
*Intercepts*							
Threshold (NT)		**2.24**	**2.10, 2.44**	**1.72**	**1.59, 1.86**	**2.09**	**1.87, 2.53**
Threshold (Praise)		**2.06**	**1.84, 2.47**	**1.70**	**1.61, 1.81**	**1.71**	**1.55, 1.93**
Threshold (Noncomp)		**1.42**	**1.29, 1.57**	**1.39**	**1.25, 1.55**	**1.74**	**1.28, 2.59**
*Regression Path Intercepts*							
Predictor at Time *t -*1	Outcome at Time *t*						
NT	NT	**0.17**	**0.04, 0.31**	**0.19**	**0.10, 0.29**	**0.23**	**0.09, 0.35**
NT	Praise	**-0.25**	**-0.53,** **-0.06**	-0.04	-0.15, 0.05	-0.01	-0.17, 0.11
NT	Noncomp	**-0.15**	**-0.35,** **-0.01**	0.02	-0.09, 0.12	-0.09	-0.56, 0.16
Praise	NT	**0.16**	**0.05, 0.30**	0.04	-0.08, 0.16	0.05	-0.12, 0.20
Praise	Praise	**-0.12**	**-0.21,** **-0.01**	0.05	-0.03, 0.14	**0.14**	**0.03, 0.24**
Praise	Noncomp	-0.03	-0.20, 0.12	**-0.23**	**-0.37,** **-0.08**	-0.17	-0.60, 0.23
Noncomp	NT	**0.12**	**0.01, 0.28**	-0.02	-0.14, 0.08	-0.07	-0.34, 0.08
Noncomp	Praise	-0.17	-0.34, 0.03	**-0.13**	**-0.21,** **-0.03**	0.04	-0.08, 0.16
Noncomp	Noncomp	**0.42**	**0.24, 0.57**	**0.16**	**0.08, 0.26**	0.11	-0.06, 0.29

Unstandardized estimates are shown

Bolded entries designate effects that are non-null based on 0 not being within the 95% credible interval

*Int* Intercept, *Var* Variance, *N* Parent negative talk, *Noncomp* Child noncompliance

**Fig. 1:**
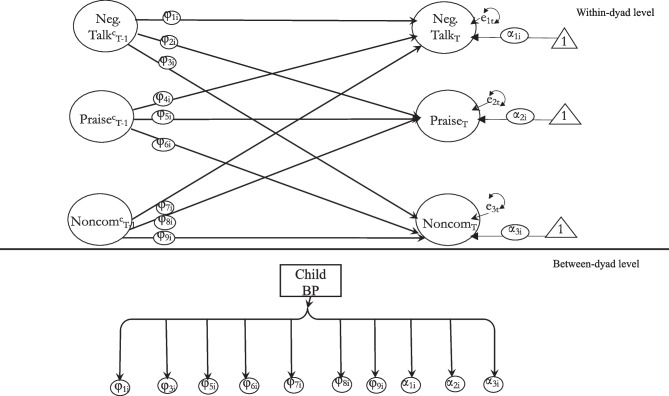
Conceptual Model. Neg Talk = Parent negative talk. Noncomp = Child noncompliance. Child BP = Child disruptive behavior problems or ADHD symptoms

We then conducted two sets of DSEMs to evaluate, per task, between-dyad differences in within-dyad processes, based on (a) child disruptive behavior problems and (b) ADHD symptoms. Our conceptual model is shown in Fig. 1. Non-null between-dyad effects are below. Estimates of between-dyad effects of child externalizing behavior problems and ADHD symptoms on within-dyad processes, for child- and parent-led play, are in Supplementary Tables 4 and 5. Estimates of between-dyad effects for the clean-up task are in Table 4.

**Table 4:** Between-dyad covariate effects for Clean-Up

Between-level predictor	Child Ext BP			Child ADHD	
Effect	Posterior Median		95% Credible Interval	Posterior Median	95% Credible Interval
α(NT) on problems	0.00		-0.02, 0.02	0.03	-0.01, 0.09
α(Praise) on problems	-0.01		-0.03, 0.01	-0.01	-0.04, 0.03
α(Noncomp) on problems	0.02		-0.01, 0.04	**0.07**	**0.02, 0.14**
φ(NT_t-1_ NT_t_) on problems	0.01		-0.01, 0.02	0.02	-0.01, 0.05
φ(NT_t-1_ Noncomp_t_) on problems	0.02		-0.01, 0.05	**0.06**	**0.02, 0.13**
φ(Praise_t-1_ Praise_t_) on problems	-0.00		-0.01, 0.01	-0.00	-0.03, 0.02
φ(Praise_t-1_ Noncomp_t_) on problems	0.02		-0.01, 0.06	0.00	-0.08, 0.10
φ(Noncomp_t-1_ NT_t_) on problems	-0.01		-0.03, 0.00	**-0.04**	**-0.09, -0.01**
φ(Noncomp_t-1_ Praise_t_) on problems	0.00		-0.01, 0.02	0.01	-0.03, 0.04
φ(Noncomp_t-1_ Noncomp_t_) on problems	-0.02		-0.03, 0.00	**-0.04**	**-0.07,** **-0.00**

Unstandardized estimates are shown

Bolded entries designate effects that are non-null based on 0 not being within the 95% credible interval

*Ext BP* Externalizing behavior problems, *NT* Parent negative talk, *Noncomp* Child noncompliance

#### Aim 1. Average Within-Person Processes

##### Within-Child Carryover

Consistent with hypothesis 1a, there was non-null positive within-child carryover in noncompliance in child- and parent-led play, such that during these tasks, changes in child noncompliance were likely to persist from one epoch to the next (Table 3). During child-led play, the unconditional probability of child noncompliance (i.e., within-child trait-like noncompliance at time *t* -1) was 11.48% but this increased to 19.95% when the child was noncompliant in the prior epoch. During parent-led play, the unconditional probability of child noncompliance was 10.85% but increased to 13.78% if the child exhibited noncompliance in the prior epoch. Contrary to expectations, there was a null carryover in child noncompliance during clean-up (Table 3).

##### Within-Dyad Cross-Lagged Processes: Parent Negative Talk and Child Noncompliance

Contrary to hypothesis 1b, during child-led play, parent negative talk *negatively* predicted their child’s subsequent noncompliance. If the parent exhibited negative talk in the prior epoch, the estimated probability of child noncompliance decreased from 11.48% to 9.22%. However, consistent with expectations, child noncompliance positively predicted subsequent parent negative talk. During child-led play, the unconditional probability of parent negative talk was 1.94% but this increased to 2.50% when the child was noncompliant in the prior epoch. Simply put, during child-led play, child noncompliance was less likely to occur following parent negative talk, but parent negative talk was more likely to occur following child noncompliance. Contrary to our hypotheses, during parent-led play and clean-up, parent negative talk did not predict subsequent child noncompliance, which did not predict parent’s subsequent negative talk (Table 3).

##### Within-Dyad Cross-Lagged Processes: Parent Praise and Child Noncompliance

Contrary to hypothesis 1c, on average, during child-led play and clean-up, parent praise did not predict subsequent child noncompliance, and child noncompliance did not predict subsequent parent praise (Table 3). However, consistent with hypotheses, during parent-led play, parent praise negatively predicted subsequent child noncompliance, and child noncompliance negatively predicted subsequent parent praise (Table 3). If their parent praised them in the prior epoch, the estimated probability of child noncompliance decreased from 10.85% to 7.57%. If their child was noncompliant in the prior epoch, the estimated probability of parent praise decreased from 5.47% to 4.27%. In other words, during parent-led play, child noncompliance was less likely to occur following parent praise, and parent praise was less likely to occur following child noncompliance.

#### Aim 2. Between-Dyad Differences in Within-Dyad Processes

Overall, estimates of the within-person and within-dyad intercepts and regression paths from models that examined between-dyad differences based on child disruptive problems (Supplementary Table 2) or child ADHD symptoms (Supplementary Table 3) were consistent with the base models that did not contain between-level predictors (Table 3). Results suggested child disruptive behavior problems and ADHD symptoms accounted for differences in the trait-like component of parent negative talk during child-led play (Supplementary Table 4). During child-led play, parents of children with low disruptive behavior problems (-1 SD below the mean) had a 1.66% unconditional probability of displaying negative talk, whereas parents of children with elevated (+ 1 SD above the mean) disruptive behavior problems had a 3.48% unconditional probability of displaying negative talk. Similarly, parents of children with fewer ADHD symptoms (-1 SD below the mean) had a 1.96% unconditional probability of displaying negative talk whereas parents of children with elevated ADHD symptoms (+ 1 SD above the mean) had a 3.45% unconditional probability of displaying negative talk during child-led play. Results also revealed ADHD mean differences in the trait-like component of child noncompliance during clean-up (Table 4), such that children with fewer ADHD symptoms (-1 SD below the mean) had a 7.64% unconditional probability of displaying noncompliance, whereas children with elevated (+ 1 SD above the mean) ADHD symptoms had a 15.34% unconditional probability of displaying noncompliance.

##### Between-Dyad Differences in Within-Child Carryover in Noncompliance

Child ADHD symptoms predicted less carryover in child noncompliance, during clean-up only (Table 4). Only children with below average (-1 SD) levels of ADHD symptoms showed carryover or persistence in child noncompliance during clean-up, Est = 0.38, 95% credible interval: [0.11, 0.65]. Children with fewer ADHD symptoms (-1 SD below the mean) had a 7.64% unconditional probability of being noncompliant; if they were noncompliant in the prior epoch, their probability of being noncompliant increased to 12.10% in the subsequent epoch.

##### Between-Dyad Differences in Within-Dyad Cross-Lagged Processes

During clean-up, child ADHD symptoms predicted within-dyad relations between parent negative talk and child noncompliance (Table 4). ADHD symptoms positively predicted the effect of parent negative talk on subsequent child noncompliance, and negatively predicted the effect of child noncompliance on subsequent parent negative talk (Table 4). Post-hoc probing at ± 1 SD mean ADHD symptoms revealed the effect of parent negative talk on their child’s subsequent noncompliance was only non-null when children had fewer ADHD symptoms, Est = -0.46, 95% credible interval: [-0.95, -0.12]. That is, parent negative talk reduced the likelihood of subsequent child noncompliance only for children with fewer ADHD symptoms. Children with fewer ADHD symptoms (-1 SD from the mean) had a 7.64% unconditional probability of noncompliance; if their parents displayed negative talk in the prior epoch, their probability of subsequent noncompliance decreased to 4.03%, a non-null difference (Supplementary Table 6). The effect of child noncompliance on their parents’ negative talk was null at low and high levels of ADHD symptoms.

## Discussion

The present study aimed to improve understanding of real-time antecedents and consequences of child noncompliance, a behavior problem theorized to arise from bidirectional relational processes (Granic & Patterson, 2006; Kalb & Loeber, 2003; Owen et al., 2012). Leveraging intensive longitudinal data on child noncompliance, collected via three unique task demands, and a novel statistical modeling approach, we evaluated theory-derived hypotheses regarding within-child carryover and within-dyad cross-lagged processes between parent behavior and child noncompliance during parent–child interactions. Given that parents’ socialization goals and the effects of parent socialization behaviors differ across conditions (e.g., child behavior, settings; Eisenberg et al., 1998; Kalb & Loeber, 2003), we evaluated within-dyad dynamics in three tasks: child-led play, parent-led play, and clean-up. Results offered mixed support for hypotheses. School-aged children’s noncompliance was predicted by *and* predicted parent behavior, but specific antecedents and consequences of child noncompliance varied. Further, within-dyad processes, especially during demanding tasks, may differ between families. During the clean-up task, only children with fewer ADHD symptoms exhibited a predictable pattern of noncompliance influenced by prior noncompliance and parental negative talk, relative to their counterparts with elevated ADHD symptoms.

## Average Within-Dyad Processes Involving Child Noncompliance

Germane to dynamic models of parent–child coercion is the initial negative reciprocation between child noncompliance and parent negativity (Granic & Patterson, 2006). Consistent with experimental evidence that aversive child behavior elicited adult negative behavior during lab-based interactions (e.g., Wymbs et al., 2015), and extending prior dynamic systems research on high-risk mothers of preschool-aged children (Geeraerts et al., 2021; Lunkenheimer et al., 2016), our results suggested that child noncompliance evoked subsequent parent negative talk during a child-led play task. However, contrary to our theory-derived expectation of bidirectional amplification of negative dyadic behavior, parent negative talk was *not* an antecedent of child noncompliance, and in fact, predicted a lower likelihood of child noncompliance. Among typical families participating in a child-led play task, brief moments of parent negativity may quickly redirect children and return the dyad to fluent, harmonious play. Reprimands and negative nonverbal parent responses, especially when paired with a command, have been associated with child noncompliance in both naturalistic and experimental studies (Owen et al., 2012). During child-led play, parents often avoid giving direct commands (e.g., “Don’t get the blocks out yet”), and therefore may use negative talk to prime their commands or as a vague “beta” command (e.g., commands that lack clear directions regarding desired behavior change; “Stop being a pest”), which may help promote child compliance in this context.

The antecedents of child noncompliance also varied according to task demands. Whereas parent negative talk arrested child noncompliance during child-led play, bidirectional relations where parent praise dampened child noncompliance, and vice versa, emerged during parent-led play. When parents lead playful interactions, they are often focused on *directing* their child’s play in ways that promote learning and intentionally socialize children’s decision-making and problem-solving, including modeling these behaviors (Zimmerman & Schunk, 2011). Following the child’s lead (e.g., monitoring, encouraging, or acknowledging the child’s effort) may be one way that parents scaffold children’s executive functioning and self-regulation (Obradović et al., 2021) while being less directive of children’s behaviors. Further, withdrawal of praise in response to noncompliance may potentiate the reinforcement value of parent praise in this context, underscoring the importance of *contingent* in-the-moment use of praise. In contrast to the mixed empirical support for the benefits of maternal autonomy support during challenging tasks (Lobo & Lunkenheimer, 2020; Lunkenheimer et al., 2017; Owen et al., 2012), contingent praise may be one way for parents to support on-task child behavior during structured play.

## Implications of Intensive Longitudinal Data

Antecedents and consequences of child noncompliance differed depending on task demands and the correlates of within-family behavioral dynamics with respect to externalizing psychopathology were also task-specific. When parents and children were faced with more challenging demands during clean-up, between-dyad differences based on child ADHD symptoms in within-dyad processes emerged. Relative to child- and parent-led play, clean-up requires more effortful control and attentional resources, as children must inhibit their responses (e.g., desire to continue playing) to effectively transition from playing to cleaning up while following parent instructions. After accounting for ADHD-related differences in initial child noncompliance, ADHD symptoms affected within-person stability and within-dyad bidirectional relations between parent negative talk and child noncompliance. For children with few ADHD symptoms, there were clear intrapersonal and interpersonal antecedents of child noncompliance, such that prior child noncompliance predicted a greater likelihood of subsequent child noncompliance whereas prior parent negative talk predicted a lower likelihood of subsequent child noncompliance. Due to executive functioning deficits (Barkley, 1997) and low frustration tolerance (Seymour & Miller, 2017), children with ADHD may find clean-up tasks taxing for self-regulation, which may be reflected in unstable intrapersonal processes.

In children with elevated ADHD symptoms, during clean-up, parent negative talk did *not* predict subsequent child noncompliance. Children with elevated ADHD may struggle to understand what is asked of them, and thus may be less equipped to detect command primes or “beta” commands implied in parent negative talk (Kalb & Loeber, 2003). Also, children with elevated ADHD symptoms, who experience repeated negative interactions with parents (McKee et al., 2004; Podolski & Nigg, 2001), may find parent negative talk more aversive and be less willing to comply with “beta” commands or command primes. By middle childhood, externalizing symptoms may reflect the *lack of coordination* between real-time changes in parent and child behavior, rather than negative moment-to-moment dyadic processes. Across development, initial negative reciprocation among at-risk families may give rise to stable, crystallized dyadic patterns (Granic & Patterson, 2006), where both parent and child are pulled toward recurring states of noncompliance and negativity, but each individual’s behavior is no longer contingent on the other’s immediately prior behavior. By examining within-dyad processes, using novel methods for disaggregating within-dyad intrapersonal and interpersonal processes from trait-like differences in overall noncompliance, we uncovered alterations in within-child carryover and between-dyad negative dynamics associated with ADHD symptoms. Future developmentally-informed work with repeated assessments of parent–child interactions must evaluate how externalizing problems are influenced by changes in real-time dyadic behavioral processes.

## Strengths, Limitations, and Future Directions

The present study benefited from strengths including intensive longitudinal data on child noncompliance and parent behaviors (negative talk and praise), collected in 10-s epochs, to elucidate within-dyad cross-lagged processes, accounting for the frequency and intrapersonal carryover in individual behavior. In contrast to most dynamic systems research that employed a unidirectional approach (see Lunkenheimer et al. (2017) for a key exception) or focused on dyadic-level processes (Granic & Patterson, 2006), evaluating the lead-lag structure of these bidirectional dyadic relations rigorously illuminated the antecedents and consequences of child noncompliance *during* parent–child interactions. Our sample of socioeconomically and ethnically diverse school-aged children addressed a critical gap in the literature, which typically focused on preschool youth when defiance is common, whereas noncompliance later in development (e.g., during middle childhood) can frequently be impairing and necessitate mental health services (Kalb & Loeber, 2003; Owen et al., 2012). Evaluation of within-dyad processes in three contexts also uncovered task-specific interpersonal antecedents and consequences of child noncompliance, depending on the “leader” of playful interactions; when faced with greater task demands during clean-up, the intrapersonal and interpersonal antecedents of child noncompliance also differed between families depending on child ADHD symptoms.

These results must also be understood in the context of study limitations and point to needed future directions. Results may not generalize to other interaction contexts, diverse caregivers or other authority figures (e.g., teachers), families with greater contextual disadvantage, different timescales (e.g., second-by-second, across development), or different developmental periods. Whereas child ADHD symptoms may be particularly salient to tasks with high cognitive demands, disruptive behavior problems may contribute to noncompliance in more relational contexts (e.g., navigating conflict; Garcia et al., 2019). Examination of co-occurring internalizing problems (e.g., anxiety) may also shed light on which children are most susceptible to coercive dynamics (Granic & Lougheed, 2016). Families with maltreatment histories may also exhibit unique behavioral patterns in structured observational tasks (Zumbach et al., 2021). Our analyses examined correlates of within-dyad dynamics among children with varying ADHD symptom levels, and future analyses with larger sample sizes are needed to examine whether these relations differ based on clinical status.

Further, results were specific to the timescale on which behavior was assessed in this study. Adjusting for intrapersonal carryover allowed us to adjust for possible continuity of behavior from one epoch to the next. However, our modeling approach did not allow us to pinpoint when children transitioned between compliant and noncompliant states, which may be achieved with shorter epoch lengths or modeling approaches such as multilevel survival analysis (Stoolmiller & Snyder, 2014). From a dynamic systems perspective (Granic & Patterson, 2006), future research is needed to evaluate whether dyadic processes have self-similar organization at different timescales (e.g., whether lack of contingency on moment-to-moment timescales leads to changes in parenting practices and worsening child behavior problems across development).

A comprehensive examination of the antecedents and consequences of child noncompliance requires assessment of parent and child behaviors, which range in frequency and intensity, as well as in experiential aspects of parent–child processes. Although the present study focused on specific parent behaviors, the *intensity* of these behaviors may influence how within-dyad processes unfold in specific contexts. For example, reciprocated dyadic negativity may require more intense or harsh negative verbalizations (Owen et al., 2012) than observed in the present study. By focusing on child noncompliance (i.e., a *response* to a parental request), we were unable to examine child-led processes that militate against externalizing problems. For example, children’s receptive and enthusiastic compliant or on-task behavior may beget positive parenting (Kochanska et al., 2015; Lunkenheimer et al., 2017) and disrupt processes that give rise to or amplify child externalizing problems, during real-time interactions or across development. Last, we employed a well-validated coding system of child and parent behavior, but parents and children may not have experienced their own and each other’s behavior as it was coded. One possibility for null effects of parent praise during child-led play and clean-up is that praise may not have been experienced as positive, but rather as controlling (Owen et al., 2012). Parents and children may have also failed to perceive behaviors noted by trained coders, as their attentional focus may be affected by task demand, setting, and the child’s clinical presentation. Studies capturing individual experience are needed to provide a nuanced understanding of dyadic processes involved in the natural ebb and flow of child noncompliance in daily life.

## Conclusion

To prevent recurring child externalizing behavior problems, we must elucidate both antecedents and consequences of child noncompliance as it occurs during parent–child interactions. The current study suggests that parents’ behavior precipitates the onset of child noncompliance. Yet, specific parental antecedents of child noncompliance differ depending on the context, highlighting ways parents can adjust how they give commands and respond to child behavior that may promote children’s well-regulated, compliant behavior. Replication of the current results would suggest that, among children with elevated ADHD symptoms, changes in negative child and parent behaviors become untethered from each other. Future research across multiple timescales, including developmental time, is needed to uncover when and why these processes emerge, and their implications for developing trajectories of externalizing disorder.

### Supplementary Information

Below is the link to the electronic supplementary material.

Supplementary file1 (DOCX 32 KB)

## Data Availability

The data and code that support the findings of this study are available from the corresponding author upon reasonable request.
