# Supplementary material for: Antecedents and Consequences of Child Externalizing Problems: Differences in Dynamic Parent–Child Processes
Source: Res Child Adolesc Psychopathol. 2023 Mar 14;52(1):7–19. doi: 10.1007/s10802-023-01045-0 (PMC10542848; doi:10.1007/s10802-023-01045-0)
Supplement: Supplementary file 1 — Supplementary file1 (DOCX 32 KB) [file 10802_2023_1045_MOESM1_ESM.docx]

**Antecedents and Consequences of Child Externalizing Problems: Differences in Dynamic Parent-Child Processes**

**Preliminary Analyses**

***Covariate selection***

Missing parent-report data. Two percent (2.1%) of the final study sample was missing data on parent-report of child externalizing behavior problems. Missingness on child behavior problems was unrelated to any potential covariates (all *p*’s > .05).

Missing micro-level observational data. Micro-level data on the parent-child interactions was not available for 92 families (39.7%) from the broader study (N = 232). Missing micro-level parent-child data was primarily due to data loss that either occurred due to audio recording failures at the time of original data collection (51.0%), or loss of original microlevel data after data collection (46.7%). Microlevel data was not usable due to errors in recording procedures for two families (2.2%). Families with microcoded parent-child interaction data (N = 140) did not differ from families with missing microcoded data (N=92) on any potential covariates or on any primary study variables (all *p*’s > .05).

Of the final study sample (i.e., the 140 families with usable micro-level data on the parent-child interaction), across all the epochs (i.e., 150 10-second epochs across the 3 interactions), 3.8% of parent praise and parent negative talk was missing due to inaudible speech or task deviation; and 68.1% of child noncompliance was missing. Regarding missingness on child noncompliance, 64.3% of child noncompliance was missing due to lack of parent command/information question and another 3.8% was missing due to inaudible speech/task deviation). Almost all (97.1%) of parents gave at least one command (range: 0-52), yielding at least one non-missing observation on child noncompliance. The number of commands parents gave was negatively associated with child age, *r* = -.228, *p* = .007, but was not associated with any other potential covariates or with any primary study variables. Associations between child age and within-level processes involving child noncompliance were all null (i.e., 95% credible interval contained 0).

Associations with primary study variables. Based on bivariate Pearson correlations, independent samples *t*-tests, and one-way ANOVAs, children’s disruptive problems and ADHD symptoms were unrelated to the other potential covariates (all *p*’s > .05).

***Between-person differences in intra-individual variability, by task***

On average, there was substantial within-person variability in each task. However, in each task, there were some people who did not exhibit change in a given behavior (e.g., who never exhibited the behavior or never stopped exhibiting the behavior).

Child-led play. During child-led play, 20% of (n=28) children exhibited constant levels of noncompliance, 20% (n=28) of parents exhibited constant levels of praise, and 37% (n=52) of parents exhibited constant levels of negative talk.

Parent-led play. During parent-led play, 14% of (n=19) children exhibited constant levels of noncompliance, 10% (n=14) of parents exhibited constant levels of praise, and 12% (n=17) of parents exhibited constant levels of negative talk.

Clean-up. During clean-up, 40% of (n=56) children exhibited constant levels of noncompliance, 33% (n=46) of parents exhibited constant levels of praise, and 45% (n=63) of parents exhibited constant levels of negative talk.

**Primary Analyses**

Supplementary Table 1.

*Estimated Within-Dyad Probabilities of Child and Parent Behavior, per Task*

|  | Child-led play | | | Parent-led play | | | Clean-up | | |
| --- | --- | --- | --- | --- | --- | --- | --- | --- | --- |
| Probability (%) | Non-compliance | Parent praise | Parent negative talk | Child noncompliance | Parent praise | Parent negative talk | Child noncompliance | Parent praise | Parent negative talk |
| Trait-like value at *t* -1 | 11.48 | 3.97 | 1.94 | 10.85 | 5.47 | 5.65 | 12.56 | 5.57 | 3.40 |
| At time *t* > 1 |  |  |  |  |  |  |  |  |  |
| Given prior child noncompliance | 19.95 | --- | 2.50 | 13.78 | 4.27 | --- | --- | --- | --- |
| Given prior parent praise | --- | --- | 2.78 | 7.57 | --- | --- | --- | 7.18 | --- |
| Given prior parent negative talk | 9.22 | 2.48 | 2.78 | --- | --- | 7.89 | --- | --- | 5.21 |

*Note*. Dashes indicate that an instance of a given behavior in the prior epoch was a null predictor of behavior in the subsequent epoch.

Supplementary Table 2.

Within-level model from model with child disruptive behavior problems as predictor

| Within-level intercepts and regression paths | | | | | | | |
| --- | --- | --- | --- | --- | --- | --- | --- |
| *Intercepts* | | | | | | | |
|  |  | Child-led Play | | Parent-led Play | | Clean-Up | |
| Effect |  | Posterior  Median | 95%  Credible Interval | Posterior  Median | 95%  Credible Interval | Posterior  Median | 95%  Credible Interval |
| Threshold (NT) |  | **2.324** | **[2.168, 2.502]** | **1.721** | **[1.592, 1.869]** | **2.109** | **[1.874, 2.633]** |
| Threshold (Praise) |  | **2.142** | **[1.871, 2.465]** | **1.689** | **[1.595, 1.799]** | **1.716** | **[1.560, 1.922]** |
| Threshold (Noncomp) |  | **1.447** | **[1.292, 1.590]** | **1.381** | **[1.248, 1.537]** | **1.717** | **[1.329, 2.284]** |
| *Regression Path Intercepts* | | | | | | | |
|  |  | Child-led Play | | Parent-led Play | | Clean-Up | |
| Predictor at  Time *t -*1 | Outcome at  Time *t* | Posterior  Median | 95%  Credible Interval | Posterior  Median | 95%  Credible Interval | Posterior  Median | 95%  Credible Interval |
| NT | NT | **0.168** | **[0.062, 0.293]** | **0.174** | **[0.082, 0.262]** | **0.234** | **[0.103, 0.365]** |
| NT | Praise | **-0.267** | **[-0.504, -0.029]** | -0.024 | [-0.131, 0.060] | -0.017 | [-0.163, 0.113] |
| NT | Noncomp | -0.135 | [-0.335, 0.007] | 0.036 | [-0.103, 0.145] | -0.112 | [-0.494, 0.174] |
| Praise | NT | **0.195** | **[0.073, 0.339]** | 0.040 | [-0.051, 0.134] | 0.021 | [-0.155, 0.164] |
| Praise | Praise | -0.148 | [-0.315, 0.008] | 0.053 | [-0.032, 0.142] | **0.135** | **[0.036, 0.241]** |
| Praise | Noncomp | 0.005 | [-0.192, 0.204] | **-0.203** | **[-0.345, -0.076]** | -0.201 | [-0.592, 0.148] |
| Noncomp | NT | **0.143** | **[0.004, 0.291]** | -0.015 | [-0.128, 0.092] | -0.062 | [-0.342, 0.110] |
| Noncomp | Praise | **-0.202** | **[-0.434, -0.066]** | **-0.112** | **[-0.209, -0.003]** | 0.043 | [-0.106, 0.177] |
| Noncomp | Noncomp | **0.422** | **[0.203, 0.583]** | 0.144 | [-0.004, 0.269] | 0.104 | [-0.099, 0.330] |

*Note.* Int = Intercept. Var = Variance. NT = Parent negative talk. Noncomp = Child noncompliance. Unstandardized estimates are shown. Bolded entries designate effects that are non-null based on 0 not being within the 95% credible interval. Between-dyad covariances and residual variances are not shown. Slope regression coefficients are in probit units.

Supplementary Table 3. Within-level model from model with child ADHD symptoms as predictor

| Within-level intercepts and regression paths | | | | | | | |
| --- | --- | --- | --- | --- | --- | --- | --- |
| *Intercepts* | | | | | | | |
|  |  | Child-led Play | | Parent-led Play | | Clean-Up | |
| Effect |  | Posterior  Median | 95%  Credible Interval | Posterior  Median | 95%  Credible Interval | Posterior  Median | 95%  Credible Interval |
| Threshold (NT) |  | **2.290** | **[2.133, 2.460]** | **1.721** | **[1.591, 1.877]** | **2.142** | **[1.920, 2.773]** |
| Threshold (Praise) |  | **2.070** | **[1.826, 2.400]** | **1.684** | **[1.590, 1.799]** | **1.710** | **[1.554, 1.906]** |
| Threshold (Noncomp) |  | **1.463** | **[1.294, 1.636]** | **1.400** | **[1.254, 1.591]** | **1.760** | **[1.402, 2.198]** |
| *Regression Path Intercepts* | | | | | | | |
|  |  | Child-led Play | | Parent-led Play | | Clean-Up | |
| Predictor at  Time *t -*1 | Outcome at  Time *t* | Posterior  Median | 95%  Credible Interval | Posterior  Median | 95%  Credible Interval | Posterior  Median | 95%  Credible Interval |
| NT | NT | **0.157** | **[0.045, 0.269]** | **0.185** | **[0.089, 0.270]** | **0.215** | **[0.058, 0.330]** |
| NT | Praise | **-0.255** | **[-0.494, -0.072]** | -0.024 | [-0.126, 0.061] | -0.002 | [-0.159, 0.139] |
| NT | Noncomp | **-0.120** | **[-0.332, -0.003]** | 0.046 | [-0.081, 0.181] | -0.201 | [-0.513, 0.035] |
| Praise | NT | **0.150** | **[0.029, 0.307]** | 0.037 | [-0.063, 0.136] | 0.005 | [-0.288, 0.159] |
| Praise | Praise | -0.104 | [-0.256, 0.052] | 0.053 | [-0.028, 0.142] | 0.116 | [-0.004, 0.225] |
| Praise | Noncomp | 0.002 | [-0.155, 0.189] | **-0.209** | **[-0.379, -0.072]** | -0.164 | [-0.458, 0.155] |
| Noncomp | NT | 0.112 | [-0.022, 0.256] | -0.025 | [-0.131, 0.082] | 0.013 | [-0.105, 0.132] |
| Noncomp | Praise | **-0.131** | **[-0.319, -0.005]** | -0.096 | [-0.198, 0.012] | -0.008 | [-0.118, 0.105] |
| Noncomp | Noncomp | **0.459** | **[0.192, 0.593]** | **0.139** | **[0.003, 0.283]** | **0.220** | **[0.035, 0.401]** |

*Note.* Int = Intercept. Var = Variance. NT = Parent negative talk. Noncomp = Child noncompliance. Unstandardized estimates are shown. Bolded entries designate effects that are non-null based on 0 not being within the 95% credible interval. Between-dyad covariances and residual variances are not shown. Slope regression coefficients are in probit units.

Supplementary Table 4.

| Between-dyad covariate effects for Child-led Play | | | | | |
| --- | --- | --- | --- | --- | --- |
| Between-level predictor |  | Child Ext BP | | Child ADHD | |
| Effect |  | Posterior Median | 95% Credible Interval | Posterior Median | 95% Credible Interval |
| α(NT) on problems |  | **0.021** | **0.005, 0.036** | **0.035** | **0.002, 0.070** |
| α(Praise) on problems |  | 0.003 | -0.013, 0.018 | 0.004 | -0.032, 0.033 |
| α(Noncomp) on problems |  | 0.006 | -0.009, 0.021 | 0.021 | -0.009, 0.054 |
| φ(NT_t-1_ 🡪 NT_t_) on problems |  | -0.003 | -0.018, 0.010 | 0.006 | -0.019, 0.034 |
| φ(NT_t-1_ 🡪 Noncomp_t_) on problems |  | -0.004 | -0.030, 0.013 | -0.001 | -0.040, 0.027 |
| φ(Praise_t-1_ 🡪 Praise_t_) on problems |  | -0.001 | -0.013, 0.014 | -0.018 | -0.041, 0.010 |
| φ(Praise_t-1_ 🡪 Noncomp_t_) on problems |  | 0.001 | -0.014, 0.021 | -0.003 | -0.035, 0.052 |
| φ(Noncomp_t-1_ 🡪 NT_t_) on problems |  | -0.011 | -0.023, 0.002 | -0.011 | -0.038, 0.020 |
| φ(Noncomp_t-1_ 🡪 Praise_t_) on problems |  | 0.017 | -0.007, 0.034 | -0.022 | -0.068, 0.013 |
| φ(Noncomp_t-1_ 🡪 Noncomp_t_) on problems |  | -0.004 | -0.021, 0.011 | -0.009 | -0.034, 0.016 |

*Note*. Unstandardized estimates are shown. Ext BP = Externalizing behavior problems. NT = Parent negative talk. Noncomp = Child noncompliance. Bolded entries designate effects that are non-null based on 0 not being within the 95% credible interval.

Supplementary Table 5.

| Between-dyad covariate effects for Parent-led Play | | | | | |
| --- | --- | --- | --- | --- | --- |
| Between-level predictor |  | Child Ext BP | | Child ADHD | |
| Effect |  | Posterior Median | 95% Credible Interval | Posterior Median | 95% Credible Interval |
| α(NT) on problems |  | 0.005 | -0.007, 0.017 | 0.011 | -0.014, 0.037 |
| α(Praise) on problems |  | 0.002 | -0.007, 0.011 | 0.003 | -0.017, 0.023 |
| α(Noncomp) on problems |  | 0.002 | -0.011, 0.015 | 0.015 | -0.015, 0.042 |
| φ(NT_t-1_ 🡪 NT_t_) on problems |  | -0.002 | -0.012, 0.007 | 0.005 | -0.015, 0.024 |
| φ(NT_t-1_ 🡪 Noncomp_t_) on problems |  | -0.008 | -0.020, 0.004 | -0.024 | -0.057, 0.006 |
| φ(Praise_t-1_ 🡪 Praise_t_) on problems |  | 0.000 | -0.009, 0.008 | -0.005 | -0.026, 0.015 |
| φ(Praise_t-1_ 🡪 Noncomp_t_) on problems |  | 0.005 | -0.009, 0.018 | 0.020 | -0.016, 0.055 |
| φ(Noncomp_t-1_ 🡪 NT_t_) on problems |  | 0.006 | -0.006, 0.018 | 0.023 | -0.002, 0.049 |
| φ(Noncomp_t-1_ 🡪 Praise_t_) on problems |  | 0.004 | -0.007, 0.017 | 0.000 | -0.025, 0.027 |
| φ(Noncomp_t-1_ 🡪 Noncomp_t_) on problems |  | 0.004 | -0.006, 0.015 | 0.003 | -0.023, 0.031 |

*Note*. Unstandardized estimates are shown. Ext BP = Externalizing behavior problems. NT = Parent negative talk. Noncomp = Child noncompliance. Bolded entries designate effects that are non-null based on 0 not being within the 95% credible interval.

Supplementary Table 6. Estimated probabilities of child noncompliance and parent negative talk, during clean-up

|  | Children with low (-1 SD) ADHD symptoms | | Children with elevated (+1 SD) ADHD symptoms | |
| --- | --- | --- | --- | --- |
| Probability (%) | Child noncompliance | Parent negative talk | Child noncompliance | Parent negative talk |
| Trait-like value at *t* -1 | 7.64 | 2.40 | 15.34 | 4.09 |
| At time *t* > 1 |  |  |  |  |
| Given prior child noncompliance | 12.10 | 4.28 | 16.48 | 2.41 |
| Given prior negative talk | 4.03 | --- | 16.25 | --- |

*Note*. Child ADHD symptoms did not account for between-person differences in within-person carryover in parent negative talk during clean-up.
